# Additional Arctic observations improve weather and sea-ice forecasts for the Northern Sea Route

**DOI:** 10.1038/srep16868

**Published:** 2015-11-20

**Authors:** Jun Inoue, Akira Yamazaki, Jun Ono, Klaus Dethloff, Marion Maturilli, Roland Neuber, Patti Edwards, Hajime Yamaguchi

**Affiliations:** 1 National Institute of Polar Research, Tokyo, Japan; 2 Japan Agency for Marine-Earth Science and Technology, Kanagawa, Japan; 3 SOKENDAI (Graduate University for Advanced Studies), Tokyo, Japan; 4 Alfred Wegener Institute, Helmholtz Centre for Polar and Marine Research, Potsdam, Germany; 5 Environment Canada, Toronto, Canada; 6 The University of Tokyo, Chiba, Japan

## Abstract

During ice-free periods, the Northern Sea Route (NSR) could be an attractive shipping route. The decline in Arctic sea-ice extent, however, could be associated with an increase in the frequency of the causes of severe weather phenomena, and high wind-driven waves and the advection of sea ice could make ship navigation along the NSR difficult. Accurate forecasts of weather and sea ice are desirable for safe navigation, but large uncertainties exist in current forecasts, partly owing to the sparse observational network over the Arctic Ocean. Here, we show that the incorporation of additional Arctic observations improves the initial analysis and enhances the skill of weather and sea-ice forecasts, the application of which has socioeconomic benefits. Comparison of 63-member ensemble atmospheric forecasts, using different initial data sets, revealed that additional Arctic radiosonde observations were useful for predicting a persistent strong wind event. The sea-ice forecast, initialised by the wind fields that included the effects of the observations, skilfully predicted rapid wind-driven sea-ice advection along the NSR.

Routine atmospheric observations within and over the Arctic Ocean are very expensive and difficult to conduct because of factors such as logistics and the harsh environment. Nevertheless, the great benefit of such observations is their enhancement of the accuracy of atmospheric circulation reproductions over the Arctic and mid-latitudes^1^ (e.g., as surface pressure and atmospheric profiles), which can then be integrated into numerical weather prediction models and atmospheric reanalysis datasets, as part of the so-called ‘data assimilation’ (DA) process. One inevitable problem with DA is the density of observations. Over the Arctic Ocean, mainly over the sea ice, observation platforms are very limited (e.g., drifting buoys^2^ and radiosondes from special research cruises^3^,^4^). Although the use of satellite observations in DA contributes to more accurate reproductions of atmospheric fields^5^, data discontinuities associated with changes in observing systems, as well as the lack of data near the pole (i.e., a data hole), are also challenging issues. Therefore, large uncertainties have been found in atmospheric reanalysis data, not just because of model performance but also because of the lack of observations over the Arctic Ocean^6^,^7^,^8^. These same problems are inherent in weather forecasts because most reanalysis is based on operational prediction systems. Aircraft observations are very effective in the prediction of severe weather events (e.g., a polar low^9^); however, their high cost excludes them from routine operational use. Radiosondes do provide fundamental atmospheric information from the surface to the stratosphere at many meteorological stations, including those in Arctic coastal regions. However, the spatial coverage of Arctic stations is much less than over the mid-latitudes. This smaller number of stations and the lower frequency of their observations must therefore be addressed to compensate the lack of data. Considering the limited financial and human resources available for conducting additional observations, an observing network optimised on a cost-benefit basis is needed for better polar predictions.

The role of additional Arctic observations has also become more important because of socioeconomic interests. Ocean states with high waves, produced by ice-free conditions^10^ and intensified wind forcing^11^,^12^,^13^, cause problems for commercial shipping routed via the Northern Sea Route (NSR)^14^,^15^. Recent colder winters over the continents are considered as part of the link between Arctic sea-ice reduction^16^,^17^ and the mid-latitude climatic system^18^,^19^, although multiple forcing factors including sea-ice loss are also considered to cause complex interactions^20^,^21^.

To evaluate the impact of additional observations on the predictability of weather and sea-ice patterns, a special radiosonde observing network was established, for a limited time in September 2013, as part of an international collaboration (the Arctic Research Collaboration for Radiosonde Observing System Experiment: ARCROSE; http://www.esrl.noaa.gov/psd/iasoa/node/123). The ARCROSE observing network consisted of the Japanese research vessel (RV) Mirai (eight launches per day), the German station at Ny-Ålesund (six launches per day), and the Canadian stations at Alert and Eureka (four launches per day; Fig. 1a). The ARCROSE period ran from 11–24 September 2013, which increased considerably the number of radiosondes launched daily (i.e., a total of 22 launches per day, compared with the usual 5) (Fig. 1c). These data were sent to the global telecommunication system (GTS) in real time and used for weather forecasts and atmospheric reanalysis datasets, with the intention that the uncertainty in the modelled atmospheric fields could be reduced by their inclusion.

From 19–21 September 2013, a high pressure system was observed along the Russian coast (Fig. 1a). The system moved westward from the East Siberian Sea to the Kara Sea. The intense pressure gradient caused strong winds along the coast. The Ostrov Kotelnyj Station (76° N, 137.9° E) recorded a 10.3 m s^−1^ daily mean surface wind speed on 20 September, whereas the RV Mirai, on the ice-free ocean (72.75° N, 168.25° W), recorded a mean wind speed from 19–21 September of approximately 11.7 m s^−1^, which caused significant upper-ocean mixing^22^,^23^. During this period, the NSR over the East Siberian Sea was partially closed by sea-ice advection and/or sea-ice formation (Fig. 1a, S1).

## Results

In this paper, we demonstrate the advantages offered by the ARCROSE data for skilful forecasting of strong winds along the NSR, using an ensemble DA system^24^ and ensemble forecasting experiments with an atmospheric general circulation model^25^,^26^,^27^. To investigate the impact of ARCROSE data on the predictability of the high pressure system, eight sets of 63-member ensemble experimental reanalysis datasets, in which the differences were manifest as combinations of ARCROSE stations, were used as the initial values for the ensemble forecasting experiments. A control reanalysis (CTL) assimilated routine global observations (NCEP PREPBUFR), which included all the ARCROSE data, while the other reanalyses were observing system experiments (OSEs) that excluded some of the ARCROSE data. The list of the daily numbers of ARCROSE data used in the CTL and OSEs is shown in Table 1. Compared with other reanalyses (e.g., the ERA-Interim^5^), this DA system has been shown to reproduce Arctic atmospheric circulations accurately^4^,^28^.

**Figure 1:**
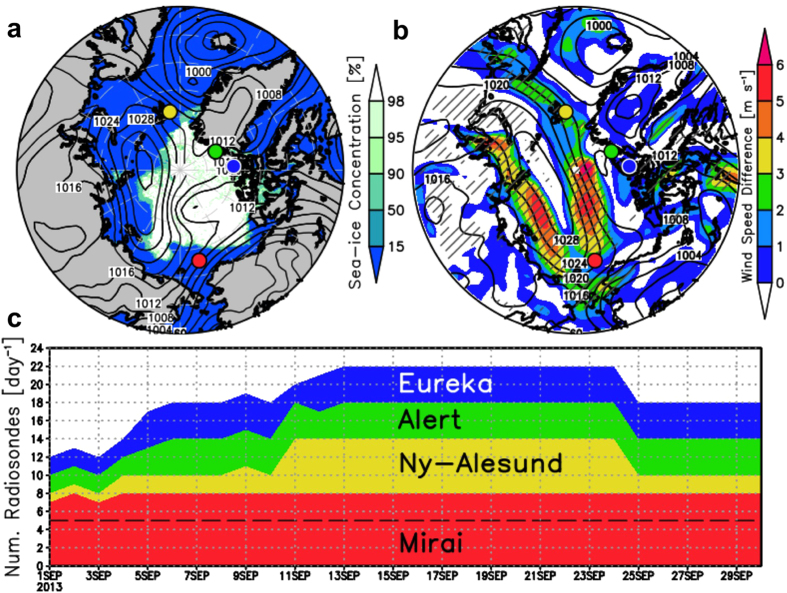
(**a**) SLP and SIC on 21 September 2013, (**b**) the difference in predicted surface wind speeds (CTL - OSE_MEAN_), and (**c**) number of radiosondes launched daily at ARCROSE stations. The ARCROSE stations are indicated by dots in (**a**,**b**) (red: RV Mirai, yellow: Ny-Ålesund, green: Alert, and blue: Eureka). Sea level pressure (SLP: hPa) and sea-ice concentration (SIC: %) data in (**a**) are taken from ERA-Interim and AMSR-2 satellite, respectively. Shading and contours in (**b**) indicate the difference in the predicted 10-m wind speeds (m s^−1^) between the CTL and OSE_MEAN_ forecasts (CTL - OSE_MEAN_), and the SLP predicted by the CTL on 21 September 2013. Hatched areas in (**b**) represent statistical significance at 99% confidence level of the shaded quantity. The dashed line in (**c**) indicates the normal number of radiosondes launched daily from operational stations (Ny-Ålesund, Alert, and Eureka). The RV Mirai remained at a fixed point (72.75° N, 168.25° W) during the ARCROSE period from 11–24 September. The Grid Analysis and Display System (GrADS) was used to create the maps in this figure.

**Figure 2:**
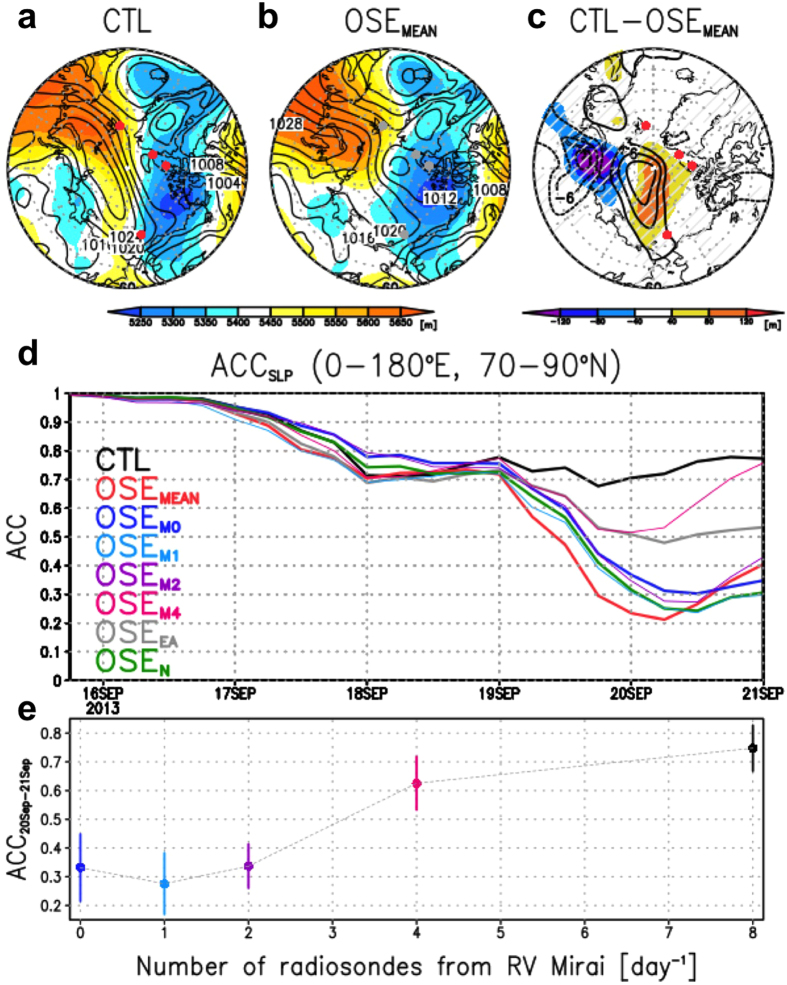
Predicted Z500 with SLP in (a) the CTL and (b) OSE_MEAN_, (c) their difference, (d) ACC for each ensemble mean forecast, and (e) ACC as a function of the number of radiosondes from RV Mirai. Ensemble mean Z500 (shading: m) and SLP (contours: hPa) at 00:00 UTC 21 September 2013 predicted by (**a**) CTL and (**b**) OSE_MEAN_. ARCROSE stations are indicated by red (grey) dots if the data are used (not used) in the initial state. (**c**) Differences in Z500 (shading) and SLP (contours) between the CTL and OSE_MEAN_. Hatched areas in c represent statistical significance at 99% confidence level of the shaded quantity. (**d**) The ACC is calculated against the climatology provided by the ERA-Interim reanalysis. Each line indicates the mean forecast for each ensemble (black: CTL; red: OSE_MEAN_; blue: OSE_M0_; cyan: OSE_M1_; purple: OSE_M2_; magenta: OSE_M4_; grey: OSE_EA_; and green: OSE_N_). The area is centred on the eastern Arctic north of 70°N. (**e**) The ACC as a function of the number of radiosondes launched daily from RV Mirai based on OSE_M0_, OSE_M1_, OSE_M2_, OSE_M4_, and CTL. Error bars indicate ±0.5 standard deviations of ACC obtained from each ensemble forecast with 63 members during the period 21–22 September. The Grid Analysis and Display System (GrADS) was used to create the maps in this figure.

The atmospheric field at 12:00 UTC on 15 September 2013 was used as the initial state. The sensitivity forecasts included OSE_MEAN_, OSE_M(0,1,2,4)_, OSE_EA_, and OSE_N_, where the subscript indicates the first letter of the ARCROSE station excluded from the dataset. The subscript number of OSE_M_ indicates the daily number of radiosondes from the RV Mirai (i.e.: OSE_M0_ used no data, OSE_M1_ used data from 12:00 UTC, OSE_M2_ used data from 00:00 and 12:00 UTC, OSE_M4_ used data from 00:00, 06:00, 12:00, and 18:00 UTC; and CTL used all the data). The difference in the 10-m wind speed between the CTL and OSE_MEAN_ was >4 m s^−1^ along the Russian coast and over the central Arctic on 21 September (Fig. 1b), which was due to the failure of predicting the high pressure system in the OSE_MEAN_ (Fig. 2b). In the CTL, the ridge at 500 hPa (Z500) extended to the Arctic Ocean across the North Pole (shading in Fig. 2a), which likely maintained the surface high pressure system (contours in Fig. 2a). In the OSE_MEAN_, in contrast, the dominant area of the Z500 ridge was limited to the Barents and Kara seas, which prevented the development of the surface anticyclonic circulation over the Russian coast (Fig. 2b). The other sensitivity forecasts (Supplementary Fig. S2a–c) were also very different from the CTL. Considering that both differences in sea level pressure (SLP) and Z500 between the CTL and OSE_MEAN_ were vertically coherent over the Laptev Sea and central Arctic (Fig. 2c), the predictability of the surface fields was most likely dependent on the forecasting skill of the upper-atmospheric levels, partly because the development of surface systems can be influenced by upper-level vortices^29^. Note that these differences are found far from the positions of the ARCROSE stations and therefore, the uncertainty in the atmospheric circulations would be caused by the growth of flow-dependent forecast errors in the upper levels^1^.

The anomaly correlation coefficients (ACCs), which indicate a pattern of correlation between the anomalies of each forecast and the control ensemble mean reanalysis^24^ from the climatology^5^, were calculated for SLP as a measure of the forecast skill (Fig. 2d). The predictive skill represented by the forecast lead time at which the ACC becomes <0.6 after 19 September was more than 2 days in the CTL (black line in Fig. 2d), while in the other forecasts, the lead time was <0.5 day. The OSE_MEAN_ (red line in Fig. 2d) appeared to have the poorest performance and the ACC fell suddenly to 0.2, simply because no ARCROSE data were used in the initial field. This timing on 19–20 September is related to the beginning of the westward movement of the high pressure system in the eastern Arctic. The OSE_N_ and OSE_M0_ followed the OSE_MEAN_ with a similar time evolution and a minimum ACC value of 0.3 (green and blue lines in Fig. 2d, respectively), while the OSE_EA_ maintained an ACC value of 0.5 during this period (grey line in Fig. 2d). The explanation for this is that each ARCROSE station has a different impact and nonlinear effect on the prediction of the phenomenon. In this case, the data from Ny-Ålesund and RV Mirai appear more influential in improving the initial state used to predict the high pressure system than those from the Canadian stations (Eureka and Alert).

To investigate the impact of observing frequency on the ACC, a relationship between the ACC and the daily number of radiosondes launched from RV Mirai is shown in Fig. 2e, based on the results from the OSE_M(0,1,2,4)_ and CTL. Although the value of the ACC becomes high as the number of radiosondes increases, the relationship is a step function rather than a linear function. The ACC is improved dramatically in the OSE_M4_ compared with OSE_M(0,1,2)_, while the difference between the OSE_M4_ and CTL is not so large. This means that a 6-hourly operation (four launches per day) is an effective observing frequency from which to receive the benefits from the additional observations. This conclusion would be also applicable to existing land stations because there is a certain difference between the CTL (6-hourly operations at Eureka and Alert) and OSE_EA_ (12-hourly operations).

**Figure 3:**
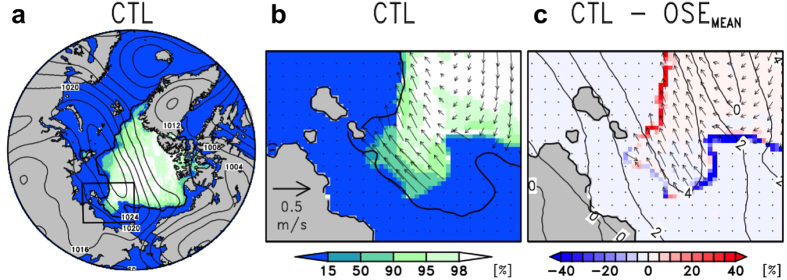
Predicted sea-ice fields in the CTL and differences between CTL and OSE_MEAN_ on 21 September 2013. (**a**) SIC (shading, %) with SLP (contours, 4-hPa interval) over the entire Arctic Ocean from the CTL. (**b**) SIC (shading, %) with sea-ice velocity (vector, m s^−1^) in the NSR (enclosed area in **a**) from the CTL. Black curves in (**a**,**b**) indicate 15% SIC contour derived from the AMSR-2 satellite. (**c**) Difference map in SIC (shading, %) with sea-ice velocity (vector, m s^−1^) and wind-speed (contours, interval 2 m s^−1^) between the CTL and OSE_MEAN_. The Grid Analysis and Display System (GrADS) was used to create the maps in this figure.

Output from an atmospheric forecast is generally used as forcing data in sea-ice forecast models, and therefore uncertainty in the output wind fields would be a source of compounding error. As shown in Fig. 1b, a large difference in the predicted wind speed between the CTL and OSE_MEAN_ would influence the sea-ice distribution because of wind-driven sea-ice drift. To assess the impact of the ARCROSE data on the sea-ice drift forecast over the NSR, we ran the ice–ocean coupled model^30^ forced by the forecast dataset from the CTL and OSE_MEAN_. The control sea–ice forecast (Fig. 3a), using the winds from CTL, was able to reproduce the observed sea-ice distribution on 21 September 2013 (Fig. 1a), particularly around the New Siberian Islands where part of the ice edge almost reached the shore (Fig. 3b). Compared with the control sea-ice forecast (Fig. 3b), the sea-ice distribution forced by the OSE_MEAN_ was underestimated by more than 20% and it remained 25 km offshore (Fig. 3c). Such a difference in the spatial extent of sea ice would have a critical influence on ship navigation because of the limited visibility over the ocean (up to a few tens of kilometres). Because wind-driven sea-ice drift is crucial in determining sea-ice conditions in this case, the wind fields derived from the inclusion of the additional radiosonde data can greatly improve the sea-ice forecast.

## Discussion

**Figure 4:**
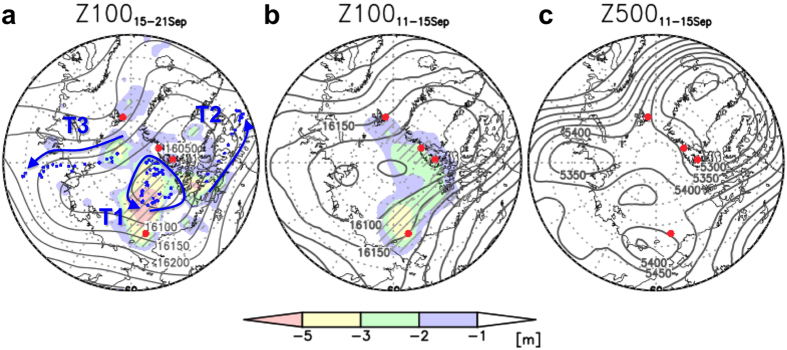
Ensemble spread differences in Z100 and Z500 between the CTL and OSE_MEAN_. (**a**) Shading indicates the difference in ensemble spread of predicted geopotential height (m) at 100 hPa (Z100_CTL_ − Z100_MEAN_) at the initial time. Blue dots are trajectories of ensemble spread difference between Z100_CTL_ and Z100_MEAN_ from the three centres of action at the initial time. Arrows depict approximate directions of trajectories. Grey contours indicate the predicted Z100 field in the CTL from 15–21 September. (**b**) As in (**a**) but the averaged condition from 11–15 September 2013 between the CTL and OSE_MEAN_ reanalyses. (**c**) As in (**b**) but at 500 hPa. Hatched areas represent statistical significance at 95% confidence level of the shaded quantity. Red dots indicate ARCROSE stations. The Grid Analysis and Display System (GrADS) was used to create the maps in this figure.

To understand flow-dependent forecast errors, in particular in the lower stratosphere and mid- troposphere, it is worth considering the impact of the ARCROSE data on the reanalysis fields before discussing the initial state (12:00 UTC 15 September) because these reanalyses are available from 11 September 2013 when the ARCROSE campaign began (Fig. 1c). The difference in the 63-member ensemble spread (CTL - OSE_MEAN_) of geopotential height at 100 (ΔZ100) and 500 hPa (ΔZ500) is shown in Fig. 4. It is clear that ΔZ100 is localised in the lower stratosphere around the ARCROSE stations (Fig. 4b), while ΔZ500 is smaller than ΔZ100 because the effect of the data assimilation of surface observations (e.g., land stations and drifting buoys) reaches the lower and middle of troposphere (Fig. 4c). This suggests that the uncertainty of a polar vortex would depend on the ARCROSE data in the reanalysis and likely the initial data. At the time of initiation on 15 September, the centres of action of ΔZ100, defined as a place where ΔZ100 has maximum value at each time step, appear to be deformed (Fig. 4a) because parts of the ARCROSE data signals prior to the initial time have been displaced around the polar vortex.

Because of the flow-dependent signals, the action centres for ΔZ100 can be tracked during the forecast period. The signal from the observations of the RV Mirai appear to be confined to a polar vortex (see contours in Fig. 4a and trajectory T1), resulting in a large uncertainty over the central Arctic. The second signal from the Canadian Archipelago travelled along Baffin Island and reached the Davis Strait (trajectory T2). This trajectory appears to be flow-dependent along Z100. The same characteristic can be seen associated with a signal from north of Franz Josef Land, which travelled southward along 90°E associated with a Z100 trough over the Laptev Sea (trajectory T3). Therefore, the region between T1 and T3 would have been the source of large uncertainty for the surface high pressure system along the NSR (Fig. 1b). This is why the skills of the OSE_N_ and OSE_M0_ were relatively low, and why the observational data from Ny-Ålesund and RV Mirai were relatively important for this event (Fig. 2d).

Although observations from research vessels are unique and useful^4^,^28^, it is unrealistic in terms of financial and human resources to conduct them in every season annually. Increasing the frequency of observations at Arctic coastal stations, instead of commissioning special observations from ships, would be useful for improving the forecast skill over and within the Arctic Ocean because the impact of additional observations is flow-dependent^31^. Considering that the variability of the jet stream associated with the wintertime polar vortex is stronger than in other seasons, the impact of additional Arctic observations on predicting extreme events in mid-latitudes might be greater during winter. The Year of Polar Prediction (YOPP) from mid-2017 to mid-2019 proposed by the World Weather Research Programme - Polar Prediction Project (WWRP-PPP) would be the best opportunity to address the issues.

## Methods

### Observations

The radiosonde observations from the ARCROSE stations during 11–24 September 2013 were obtained using Vaisala RS92 radiosondes. The launch times were 00:00, 03:00, 06:00, 09:00, 12:00, 15:00, 18:00, and 21:00 UTC at RV Mirai; 00:00, 06:00, 09:00, 12:00, 15:00, and 18:00 UTC at Ny-Ålesund; and 00:00, 06:00, 12:00, and 18:00 UTC at Alert and Eureka. All ARCROSE data were entered into the GTS and >99% of the data were found in the NCEP PREPBUFR Global Observation dataset, suggesting that ARCROSE data had been used in routine weather forecasts and reanalysis data.

### Data assimilation system (ALEDAS2)

The AFES-LETKF experimental ensemble reanalysis version 2 (ALERA2) dataset, produced with AFES-LETKF data assimilation system 2 (ALEDAS2^24^), was used as the reference reanalysis (CTL). ALEDAS2 includes the Atmospheric General Circulation Model (AGCM) for the Earth Simulator (AFES^25^,^26^,^27^) for forecasts and the local ensemble transform Kalman filter (LETKF^32^,^33^) for analysis. In the forecast step of ALEDAS2, an ensemble forecast with 63 members is conducted with AFES with a horizontal resolution of T119 (triangular truncation with truncation wave number of 119, 1° × 1°) and vertical levels L48 (sigma-level, up to approximately 3 hPa). The sea surface temperature and sea-ice concentration data were obtained from the National Oceanic and Atmospheric Administration (NOAA) daily 1/4° Optimal Interpolation Sea-Surface Temperature (OISST) version 2^34^. In the analysis step, observations were assimilated into the ensemble forecast with LETKF, and the observations were prepared from PREPBUFR datasets compiled by the National Centers for Environmental Prediction (NCEP) and archived at the University Corporation for Atmospheric Research (UCAR). The data assimilation window was set to 6 hours. Eight reanalysis sets were made to provide the initial data for the forecasting experiments described below (CTL, OSE_MEAN_, OSE_M(0,1,2,4)_, OSE_EA_, and OSE_N_).

### Forecasting experiments

We applied the method from ref. 28 using AFES as the forecast model that had the same model description as ALEDAS2 (a spectral, Eulerian, and primitive-equation AGCM^26^ with T119L48). Therefore, the forecast results could be directly compared with the CTL. The ensemble reanalysis CTL or OSE was used for the initial values in the experiments; i.e., the forecast experiments were types of ALEDAS2 in which the data assimilation procedure was off-line. The atmospheric fields of the CTL and OSE at 12:00 UTC on 15 September 2013 were used as the initial values for the forecasting experiments. A 10-day integration was performed in the experiments. There were 63 ensemble members used for the forecasts with the same members for the reanalyses of CTL and OSE (Table 1). Similar results were obtained when other initial states were used (e.g., 00:00 UTC on 15 and 16 September 2013), suggesting that the sensitivity to initial states was small.

### Ice–ocean coupled model

We used an ice–ocean coupled model^30^ where the ocean component is based on a general coordinate version of the Princeton Ocean Model^35^. The model domain comprised the entire Arctic Ocean with a horizontal resolution of approximately 25 km and a vertical grid of 33 sigma levels. The model was initialised using the monthly mean temperature and salinity from Polar Science Center Hydrographic Climatology (PHC)^36^, without ocean circulation or sea ice. After a spin up for 10 years using 6-hourly wind stress and heat fluxes calculated from the ERA-Interim data from 2000 and the monthly mean discharge of 13 rivers (AOMIP), the model was integrated from January 2001 to September 2013. Simulated oceanic fields were used as the initial conditions in the present experiments with the initial time as 12:00 UTC 15 September 2013. The initial sea-ice concentration was obtained from satellite observations (Supplementary Fig. S1) and its thickness estimated using an algorithm^37^. Sea-ice velocity was set to zero under the assumption that the sea ice responds quickly to wind stress. The drag coefficient between the atmosphere and sea ice was set to 3.7 × 10^–3 ^38 assuming a marginal ice zone. The sea-ice thermodynamic processes were turned off to ignore the impact of the sensitivity of the initial conditions of an upper-ocean structure on sea-ice melting and growth. The model was integrated from 12:00 UTC 15–24 September 2013 using the CTL and OSE_MEAN_ forcing.

## Additional Information

**How to cite this article**: Inoue, J. *et al.* Additional Arctic observations improve weather and sea-ice forecasts for the Northern Sea Route. *Sci. Rep.* **5**, 16868; doi: 10.1038/srep16868 (2015).

## Supplementary Material

Supplementary figures

## Figures and Tables

**Table 1:**
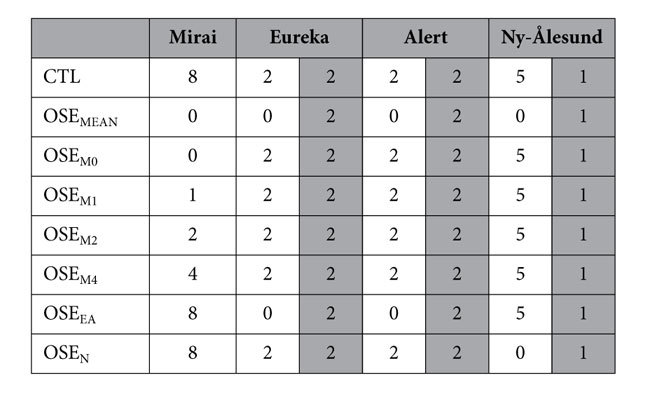
Daily number of ARCROSE data used in the reanalyses and forecasts.

The numbers in the grey columns indicate the number of routine observations. Subscript of OSE indicates the first letter of ARCROSE stations excluded from the data. Subscript number of OSE_M_ indicates the number of observations from RV Mirai used in the reanalyses and forecasts.
