# Supplementary material for: Additional Arctic observations improve weather and sea-ice forecasts for the Northern Sea Route
Source: Sci Rep. 2015 Nov 20;5:16868. doi: 10.1038/srep16868 (PMC4653624; doi:10.1038/srep16868)
Supplement: Supplementary figures [file srep16868-s1.pdf]

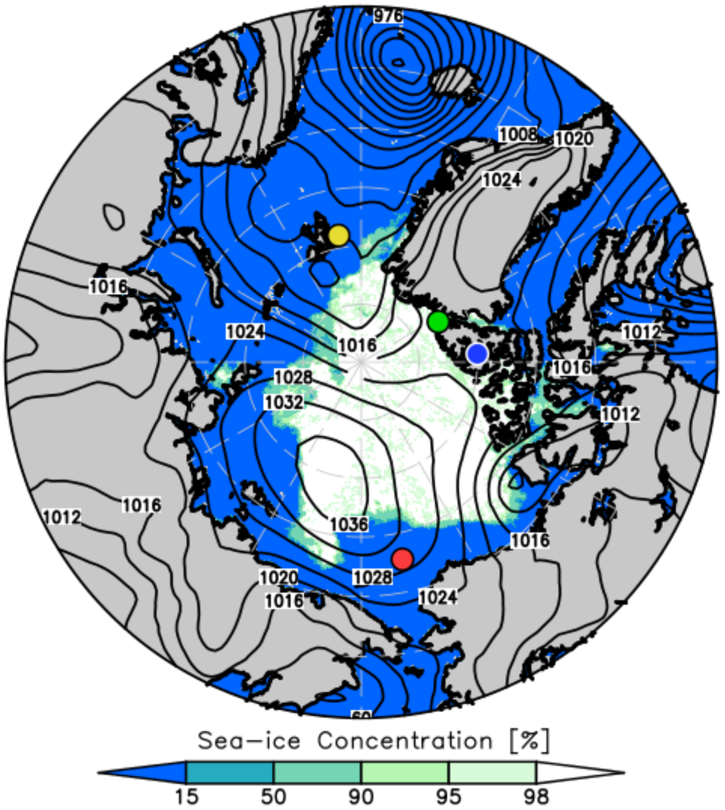

Figure S1 | SLP and SIC on 15 September 2013.

The ARCROSE stations are indicated by dots (red: RV Mirai; yellow: Ny-Ålesund; green: Alert; and blue: Eureka). Sea level pressure (SLP: hPa) and sea-ice concentration (SIC: %) data were taken from ERA-Interim and AMSR-2 satellites, respectively. The Grid Analysis and Display System (GrADS) was used to create the map in this figure.

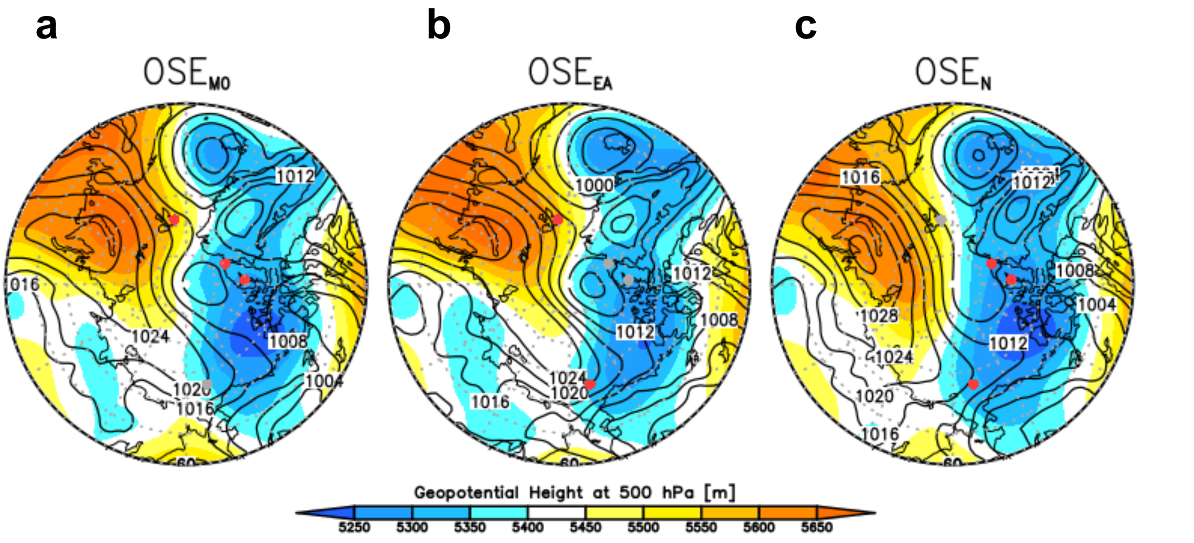

Figure S2 | Predicted Z500 with SLP.

Ensemble mean Z500 (shading: m) and SLP (contours: hPa) at 00:00 UTC 21 September 2013 predicted by (**a**) OSE$_{M0}$, (b) OSE$_{EA}$, and (**c**) OSE$_{N}$. ARCROSE stations are indicated by red (grey) dots if the data are used (not used) in the initial state. The Grid Analysis and Display System (GrADS) was used to create the maps in this figure.
